# Learning at a social distance: A medical student telehealth service for COVID-19 patients

**DOI:** 10.15694/mep.2020.000249.2

**Published:** 2021-09-22

**Authors:** Steven Server, Sophia Uddin, Matthew GoodSmith, Annie Zhang, Renslow Sherer, Jonathan Lio

**Affiliations:** 1 University of Chicago

**Keywords:** Undergraduate Medical Education, COVID-19, Ambulatory Care, Telemedicine

## Abstract

This article was migrated. The article was marked as recommended.

COVID-19 has disrupted traditional forms of clinical practice in both inpatient and outpatient settings. This novel, potentially-fatal infection proliferated to such a degree that many patients with mild disease had to engage in self-care at home. This disruption to clinical services has also upended in-person clerkship education across the country, leading to sustained periods of student furloughing.

We developed a telehealth service-learning opportunity for COVID-19 patients who were advised to self-care in their homes. The service was staffed by medical students in their clinical training years, providing triage advice to patients, their families, and co-habitants until their symptoms improved. Callers set patient education around red flag symptoms as their first priority, but also offered counsel on home infection control and self-isolation strategies, composed work letters, offered resources regarding home management issues such as food and sanitation, and attended to the mental health needs of the patients and their families. An attending was on-call daily to assist and educate students about issues relating to clinical decision-making and the social determinants of health. A survey assessed medical students’ opinions on the service.

Student respondents found the service valuable, with 100% agreeing or strongly agreeing that the service was worth their time and important. Respondents reported learning important telehealth skills such as triage and patient education. Overwhelmingly, students found emotional connections with patients to be the most meaningful aspects of the service.

Our telehealth service allowed students to learn from patients in a longitudinal manner, while remaining safely away from clinical settings. This service may prove a useful model for others in the case of another outbreak, particularly when medical students are furloughed. We hope to develop more clinical experiences in telehealth for medical students moving forward.

## Introduction

On April 14, the American Association of Medical Colleges (AAMC) “strongly suggest[ed] that medical students not be involved in any direct patient care activities” in COVID-19 hotspot areas, due to limited amounts of personal protective equipment and undue exposure for students, people in their social circles, and patients themselves ( Whelan *et al.*, 2020). This state of affairs affected medical schools across the nation. From March 16 until June 8, 2020, medical students at the University of Chicago (UChicago) were released from clinical clerkship responsibilities due to COVID-19. In the era of COVID-19, medical educators and students alike were confronted with a dearth of in-person clinical learning opportunities that are an essential part of medical education. Many of the traditional service structures in both inpatient and outpatient contexts have been replaced with telehealth practice.

In March and April, COVID-19 cases exploded across the country, and UChicago Medicine saw precipitous increases in the number of patients at its Emergency Department and its drive-thru testing facility presenting with symptoms of Influenza-like illness (ILI) with concern for COVID-19. The majority of COVID-19 patients did not need inpatient care, but were thought to benefit from regular follow-up throughout their recovery, given the rapidity with which COVID-19 cases have been seen to clinically deteriorate. Even those with stably mild disease were thought to benefit from regular check-ins: it was desirable that patients and their families, confronted with a novel, potentially-fatal illness, and isolated at home, should have a familiar contact to manage any confusion or distress. The vast number of these mild COVID-19 cases, however, meant that comprehensive follow-up posed a logistical challenge for the community’s healthcare workers. This situation only became more challenging as the State of Illinois and UChicago Medicine ramped up their testing capacity and the pandemic progressed.

Since 2014, the American Medical Association (AMA) has encouraged the U.S. House of Representatives to support telehealth programs, as it is thought that telehealth may improve issues of access to care, particularly for rural populations ( AMA, 2014). Officials and clinicians alike have advocated for expanding educational opportunities in telehealth. In 2016, the AMA adopted a policy in its annual meeting which encouraged institutions such as the Liaison Committee on Medical Education (LCME) and the Accreditation Council for Graduate Medical Education (ACGME) to include telehealth as a core competency in both undergraduate and graduate medical education ( AMA, 2016).

Telehealth had previously been seen as a desirable adjunct to in-person practice, but it quickly became indispensable under a regime of social distancing in the era of COVID-19 ( Hollander and Carr, 2020). On May 14, 2020, the AAMC sent an open letter to Centers for Medicare and Medicaid Services (CMS) voiced support for sustained telehealth resources even once the pandemic tide abates ( Orlowski, 2020). Our contribution to mitigate the disruption to in-person clinical learning for medical students, while also addressing the massive number of patients caring for themselves at home with an unpredictable new infectious disease, was to develop a telehealth service-learning opportunity for medical students.

## Intervention

In early April, a recruitment note went out to medical students in their clinical training years asking for volunteers to lead the hospital’s COVID-19 follow-up service. Three student leaders volunteered and formed three teams of approximately 12 students each. Coordinating attendings from the Section of Infectious Diseases organized an online orientation to discuss common symptoms, red flag symptoms, and proposed workflow norms for the service. A call structure was developed for the distribution of new patients. Each morning, the student leaders on call searched an electronic medical record (EMR) list populated with patients who received notification of a positive SARS-CoV-2 test. That leader distributed new patients on that list equally among their team members, and student volunteers followed with those patients until designated “complete”. This service structure, rather than a shift-based service, was selected to allow for a more longitudinal relationship with patients.

On the first call with a patient, volunteers reviewed the EMR and took a targeted history related to the patient’s COVID-19 course. On this first call, in addition to history-taking meant to better risk-stratify patients (history of hypertension, lung disease, obesity, other conditions associated with moderate/severe COVID-19), patient education was offered, outlining up-to-date guidelines for effective self-isolation, safe use of anti-pyretics, and most importantly, red flag symptoms which would prompt a visit to the ED. In addition, volunteers also inquired about patient access to food, prescription drugs, safe isolation housing, transportation, and primary care follow-up. If a patient was in need of one or more of these, volunteers connected patients to phone and digital resources offered by the hospital, community, or City of Chicago.

Following the first call, students called patients every 24 to 48 hours, depending upon the severity of a patient’s symptoms. If a patient reported red flag symptoms, or if a volunteer had concern about moderate/severe disease, volunteers discussed with the service’s attending, and referred the patient to the ED for further care. Patients continued to receive calls from volunteers until they were either hospitalized or improving on two consecutive calls. At that time, the patient was again reminded about red flag symptoms and the hospital’s COVID-19 resource hotline and designated complete.

The service structure was flexible enough to adapt to changing conditions as the pandemic progressed. As the patient volume increased in mid-April, due to increases in both incidence of COVID-19 and in number of tests performed by our hospital, this effort was scaled by adding an additional team leader and group of volunteers. The initial every three day call schedule without weekend coverage was transitioned to an every four day, weekends-included schedule.

## Outcomes

Volunteers followed up with patients for an average of 4.5 days, with a range of 0 days (patient asymptomatic on first call) to 30 days of follow-up. Students’ perspectives on the program were assessed with a 16-item online survey administered from 5/15/20 to 5/22/20. Participants rated their level of agreement with statements about the program using a four-point Likert scale (strongly disagree, disagree, agree, strongly agree). They also described their experiences in a free response section. Forty-two of 53 (79%) volunteers responded to the survey. Results can be seen in Table 1.

**Table 1:** Survey results (n=42)

			RESPONSE		
	STATEMENT	Strongly Agree	Agree	Disagree	Strongly Disagree
1	This volunteer experience was valuable to me in the context of my medical training.	27 (64%)	15 (36%)	0	0
2	I made a meaningful contribution to patient care through this volunteer experience.	31 (74%)	11 (26%)	0	0
3	I am more knowledgeable about patient symptoms of COVID-19 as a result of this volunteer experience.	31 (74%)	10 (24%)	1 (2%)	0
4	I feel more confident to answer questions about COVID-19 management as a result of this volunteer experience.	26 (62%)	14 (33%)	2 (5%)	0
5	I understand the patient experience of COVID-19 better as a result of this volunteer experience.	34 (81%)	8 (19%)	0	0
6	I have a better understanding of psychosocial factors affecting patients in the COVID-19 pandemic.	35 (83%)	6 (14%)	1 (2%)	0
7	As a result of this volunteer experience, I feel a stronger sense of connection to the patients in my community.	32 (76%)	9 (21%)	1 (2%)	0
8	Medical schools should provide training in telehealth.	28 (67%)	13 (31%)	1 (2%)	0
9	After this experience, I feel more comfortable with evaluating patients over the phone.	12 (29%)	27 (64%)	3 (7%)	0
10	After this experience, I feel more confident to determine whether a patient needs to go to the ED.	13 (31%)	22 (52%)	7 (17%)	0
11	I had adequate mentoring and support from faculty.	23 (55%)	17 (40%)	2 (5%)	0
12	This volunteer experience was well-organized.	33 (79%)	9 (21%)	0	0
13	Expectations were made clear for volunteers.	32 (76%)	10 (24%)	0	0
14	My questions were answered in a clear and timely fashion.	37 (88%)	5 (12%)	0	0

Respondents found the service worthwhile and consequential. Every respondent agreed or strongly agreed that the program was valuable to their medical training, and 100% of respondents agreed or strongly agreed that they made a meaningful contribution to patient care as a result of their participation, with nearly 75% strongly agreeing (questions 1 and 2). Respondents also reported that they learned important telehealth skills as a result of their time on the service: 93% of respondents either strongly agreed or agreed that they felt more comfortable evaluating patients over the phone (question 9), while 83% strongly agreed or agreed that they felt more comfortable triaging patients for an ED visit over the phone (question 10).

Respondents reported that learning on the service transcended clinical skills. Over 81% of respondents strongly agreed that they acquired better insight into patient experience of COVID-19, and over 75% of respondents strongly agreed that they felt a stronger sense of connection to patients in the community (questions 5 and 7). All participants supported this survey item with free responses offering descriptions of their most meaningful calls. Several common themes emerged in their responses: these can be seen in Supplementary File 1, with representative quotes. In general, volunteers found the program allowed them to help others and feel useful “at a time of great sorrow and uncertainty.”

One hundred percent of participants found that the program was well-organized, with clear expectations, and 95% agreed or strongly agreed that there was adequate mentoring from attendings. Data regarding the clinical outcomes of the service is described by our group in a separate report.

## Discussion

COVID-19 has changed many areas of life, not least medical education. In the literature, we saw full-throated defenses for creative approaches to support undergraduate medical education through the pandemic ( Newman and Lattouf, 2020). Our creative strategy to mitigate these disruptions was to develop a telehealth program to allow clinical learning to continue at a distance, while delivering essential care to patients facing a novel, ill-described disease.

Our COVID-19 follow-up telehealth service allowed for students to learn from patients in a longitudinal manner, while allowing students to remain safely away from hospital environments. Volunteers felt they developed important clinical skills in telemedicine, across a variety of domains, such as communication skills, patient education, and issues of clinical judgement, such as telephone evaluation and triage. These skills are consistent with the telehealth competencies proposed by the AAMC for medical education. We felt that we were able to accomplish our goal of creating a useful opportunity for medical students to develop their clinical skills in the setting of social distancing. This runs consistent with previous studies which have shown that medical students find telehealth education interesting, clinically useful, and a good use of their time, in both primary care and sub-specialty settings ( Dzara *et al.*, 2013; Jonas *et al.*, 2019).

Beyond developing clinical skills, however, students found emotional connections with patients to be among the most meaningful aspects of the service. As richly described in student quotes, volunteers were able to establish real emotional connections by virtue of their longitudinal relationships with patients and their families. Students felt they better understood the social determinants of health as they vividly played out in the course of a relationship with a patient, a competency that is often difficult to acquire with conventional inpatient or outpatient education. Despite the theoretical concern that telehealth could weaken the doctor-patient relationship due to lack of physical proximity, our volunteers reported feeling closer to members of their community, able to feel some element of the frustration and anxiety our patients experienced navigating the institutional and emotional landscape created by COVID-19.

We received a great deal of positive feedback from patients, who often felt shell-shocked by the phone call notifying them of their COVID-19 status, and appreciated the opportunity to have questions fielded and resources offered by a familiar voice. As such, in addition to the clinical service provided to our patients, we should not understate the importance of the psychological and emotional care provided to our patients and their families by this program.

Certainly, the service was not without its limitations, given the volatility and unpredictability of COVID-19. Out of necessity, the program was quickly arranged, with limited training. Students were occasionally required to perform tasks, such as triage, with only indirect supervision. This demanded flexibility on the part of the volunteers. The service itself had to evolve and change with the changes in the trajectory of the pandemic, making standard-setting difficult. Finally, the service had an expected time limitation, given the need for students to return to clinical clerkships, even as the pandemic continues. In general, calls to home-bound patients are being transitioned to their primary care provider as students return to clerkships.

Nevertheless, in spite of these limitations, this service may prove a useful model for others in the case of another outbreak here in the United States, or other international settings, particularly when medical students are furloughed. As the pandemic continues, we have included this telehealth service as a community service supplement to the family medicine clerkship. We are also exploring developing this service as a clinical elective as well. When pandemic conditions abate, we must also consider how this telehealth service may serve as a model for future telehealth educational opportunities at our institution. This would be consistent with the wishes of practicing physicians, medical students, and indeed, our patients.

## Take Home Messages

• COVID-19 fundamentally altered service structures in the hospital, meaning that many patients had to recover at home.
• COVID-19 also altered the ability of medical students to learn in hospital environments.
• We created a service in which medical students furloughed at home engaged in telehealth outreach to patients convalescing at home.
• Medical students found the endeavor education and worthwhile, and particularly appreciated making emotional connections to patients over the phone.
• The service is widely applicable both in the setting of future outbreaks and in traditional clerkship structures to provide students greater experience with telehealth.

## Notes On Contributors

**Steven Server**, MSc is an MD/PhD candidate at the Pritzker School of Medicine, University of Chicago.

**Sophia Uddin**, PhD is a 4th year MD candidate at the Pritzker School of Medicine, University of Chicago.

**Matthew GoodSmith** is a 4th year MD candidate at the Pritzker School of Medicine, University of Chicago.

**Annie Zhang** is a 4th year MD candidate at the Pritzker School of Medicine, University of Chicago.

**Renslow Sherer**, MD is a Professor of Medicine in the Section of Infectious Diseases and Global Health, Pritzker School of Medicine, University of Chicago. ORCID ID: https://orcid.org/0000-0003-3780-2675

**Jonathan Lio**, MD is an Assistant Professor of Medicine in the Section of Infectious Diseases and Global Health, Pritzker School of Medicine, University of Chicago. ORCID ID: https://orcid.org/0000-0002-3723-2767
