# Supplementary material for: Learning at a social distance: A medical student telehealth service for COVID-19 patients
Source: MedEdPublish (2016). 2021 Sep 22;9:249. Originally published 2020 Nov 9. [Version 2] doi: 10.15694/mep.2020.000249.2 (PMC10697485; doi:10.15694/mep.2020.000249.2)
Supplement: Peer review history

# Reviewer report: Reviewer response for version 2

**DOI:** 10.21956/mep.20284.r31495

**Published:** 2021-10-08

**Authors:** Tharin Phenwan

**Affiliations:** 1 University of Dundee

This review has been migrated. The reviewer awarded 3 stars out of 5 An interesting article and is relevant to us all given the current situation with COVID-19. Telehealth service will definitely stay with us for the time being.The content is well-structured and easy to follow. The discussion was relevant to the scope of this work. I had a few inquiries and suggestions though:-How were the 16 items in the online-survey created? Was it from the literature?-You used some of the volunteers' feedback (Supplementary file 1). Did you get their consent? Was this work exempted from the IRB? Why or why not? This needs to be stated.P5:-Themes do not 'emerge'. It could be shared meanings found within the texts but they do not emerge. I highly recommend checking Braune & Clarke article on Reflexive TA. -How did you support this claim that they emerged? What was the analytical process? Was the comments collated together? This needs to be further expanded in the texts.Discussion-Some words used in this section are values and assumptions-laden e.g. 'richly describe', 'real emotional connection', 'virtue'. I would suggest rephrase these terms. ('Show, don't tell'. Let the readers be the judge of that.)-What are the future implications for this work?

Reviewer Expertise:

NA

No decision status is available

# Reviewer report: Reviewer response for version 2

**DOI:** 10.21956/mep.20284.r31494

**Published:** 2021-09-23

**Authors:** Ken Masters

**Affiliations:** 1 Sultan Qaboos University

This review has been migrated. The reviewer awarded 4 stars out of 5 The authors have addressed the concerns that I raised in my commentary of Version 1 of the article. Although most of the changes were merely structural, they have led to a paper that flows more easily, is a worthwhile read, and is a useful contribution to the field. Well done.

Reviewer Expertise:

NA

No decision status is available

# Reviewer report: Reviewer response for version 1

**DOI:** 10.21956/mep.19143.r27675

**Published:** 2021-02-18

**Authors:** Richard Hays

**Affiliations:** 1 James Cook University

This review has been migrated. The reviewer awarded 4 stars out of 5 This article reports an interesting innovation that aims to fill some gaps in clinical experience of medical students. 2 years ago many would have thought this not very useful, but the skewed case mix in health care settings during the pandemic and changes to service configuration have us now looking for different ways of providing quality care and education. While I liked this idea, the method would have been stronger if students were to speak by phone or video with ambulatory patients have follow up for a much wider range of medical conditions. To the extent that it is possible to conduct normal business, this approach might be better for both patients and learners. Most clinicians are now more familiar and more comfortable with telehealth, so the students would certainly gain those skills as well as practice in communication skills, but the extent of clinical learning outside of Covid-19 conditions may be limited. The article would merit more stars if it included a table listing the clinical conditions covered during the telehealth sessions. It would be great to see a follow up study with telehealth for more routine initial consultations and then review clinics conducted by telehealth, again with descriptions of the clinical conditions. This method is worthy of more research.

Reviewer Expertise:

NA

No decision status is available

# Reviewer report: Reviewer response for version 1

**DOI:** 10.21956/mep.19143.r27674

**Published:** 2021-02-02

**Authors:** Ken Masters

**Affiliations:** 1 Sultan Qaboos University

This review has been migrated. The reviewer awarded 3 stars out of 5 An interesting paper about a medical student telehealth service for COVID-19 patients. Although the paper is an interesting read, there are a few things that could be addressed:• For easier reading, the Abstract block of text could be broken into paragraphs, following the structure of the paper. • The opening four sentences of the paper appear to be in an incorrect order, and need to be re-arranged (as it stands, it appears that the events at UChicago affected the entire nation, and led to the AAMC’s recommendation. ) • As medical school curricula are structured differently around the world, it would be useful if there could be some discussion of the students previous training. Simply saying that they are third- and fourth year students does not really give insight into their knowledge and possible experience, especially with exposure to patients.• Similarly, as “q3” and “q4” are not universal terms, these should be explained.• The text following Table 1 appears to introduce new data. If this is so, then raw figures as well as percentages should be given. If the text is combing data from Table 1 (e.g. the comments about triage?) then the sources (questions) of those combinations should be clarified. If it is merely repeating what is in Table 1, then this should not occur.• Some of the literature currently introduced in the Discussion (virtually the entire second paragraph of the Discussion, apart from the first sentence of that paragraph) should first be introduced in the Introduction, and then the results discussed in the Discussed in light of the previously-cited literature.So, a good read, but strengthening is necessary. I look forward to Version 2 of the paper in which these issues are addressed.

Reviewer Expertise:

NA

No decision status is available
